# Pit latrine fill-up rates: variation determinants and public health implications in informal settlements, Nakuru-Kenya

**DOI:** 10.1186/s12889-019-6403-3

**Published:** 2019-01-15

**Authors:** Fredrick Owino Gudda, Wilkister Nyaora Moturi, Omondi Steve Oduor, Edward Wanee Muchiri, Jeroen Ensink

**Affiliations:** 1 Department of Environmental Science, Faculty of Resource and Environmental Science, Egerton University, Nakuru, Kenya; 2 Department of Biological Sciences, Faculty of Science, Egerton University, Nakuru, Kenya; 3 Department of Civil and Environmental Engineering, Faculty of Engineering, Egerton University, Nakuru, Kenya; 4 Faculty of Infectious and Tropical Diseases, London School of Hygiene and Tropical Medicine, Bloomsbury, UK

**Keywords:** Shared pit latrines, Basic sanitation, Faecal sludge, Developing country

## Abstract

**Background:**

Pit latrine operational management and sludge accumulation rate, presents a challenging sanitation problem in low-income urban settlements. However, these challenges have been under-researched. This study was carried out between December 2014 and September 2015 in Nakuru, Kenya. Its objectives were to determine pit latrine management activities and content accumulation rates.

**Methods:**

A longitudinal design was used to study 100 households and their respective pit latrines. Sludge accumulation in 73 pit latrines was monitored for 10 months using a digital laser range-finder. Data analysis included normality testing and descriptive statistics. Differences in fill up across and within the study areas were analysed using one-way analysis of variance and the Fisher’s Exact Test used to determine areas with significant differences.

**Results:**

Sixty-one percent of the pit latrines were used as solid waste disposal points while 45% of the respondents had no hygiene awareness. The annual fill-up rate and individual sludge contribution were 0.87 ± 0.20 m^3^ and 41.82 l respectively. The sludge accumulation rates across the study areas had statistically significant mean differences (*p* < 0.05).

**Conclusion:**

Operational management and design affect the fill-up rates and post fill-up management operations. This study argues for a need to link information and awareness to users, construction artisans, property owners and local authorities on appropriate vault volumes and management practices. Linking the variables would ensure efficient sanitation service delivery and public health protection.

## Background

Globally, 2.4 billion people do not have access to improved sanitation services despite the gains documented during the Millennium Development Goals (MDGs) project [1]. There are significant gaps in regards to services offered to rural and urban populations, gender inequalities and exclusion of the poor from sanitation opportunities [2]. Open defecation is still being practiced by almost one billion people, hence the need to address these gaps. According to JMP 2015, the least developed countries did not meet sanitation targets with only 27% of their current populations gaining access to improved sanitation. A large number of low-income settlement dwellers rely on on-site sanitation, especially pit latrines that generate a mixture of solid and liquid wastes referred to as faecal sludge ‘FS’ [3]. This information can support sustainable Faecal Sludge Management (FSM) programs, such as emptying cycles and budgetary allocations [4, 5]. The world has recognized the urgency and need for sanitation as a strategy to combat environmental health issues. Therefore, new targets were developed under sustainable development goals (SDGs). The targets focus on ensuring availability and sustainable management of water and sanitation for all. Evidence-based policies and programs must be directed towards marginalized populations such as under-served settlements [6].

Proper sanitation services have a fundamental role in improving people’s health, economic stability, dignity, and protection of the local environment [7]. Adequate and safe sanitation supports good health and prevent disease outbreaks [8]. Proper human excreta disposal has greater importance than provision of safe water since it significantly lowers the possibility of faecal contamination of environmental resources. Moreover, appropriate human waste disposal controls the spread of diseases and minimizes transmission of water-related diseases [9]. FSM processes have significant challenges causing public health and environmental risks. If there is no proper post fill-up management plan, then the ‘FS’ remains in poorly designed pit latrines or gets unauthorized discharge into waterways, open drains and insanitary landfills [10].

The unpredictable nature of operational management of facilities poses economic and environmental sanitation constraint to users, property owners and the government [11, 12]. Theoretically, stabilization or content leaching should be equal to the sludge accumulation rates. However, with addition of non-faecal materials into the vault and low degradation of FS, the pit latrines eventually become full. They are not useful when full.

Access to improved sanitation facilities in Kenya from 1990 to 2015 has increased from 25 to 30%. However, the change is considered as limited or an indication of no progress according to the JMP classification. Rural and urban populations in the country have different sanitation challenges [13]. Approximately 60% of the urban dwellers live in informal settlements characterized by inadequate water supply and sanitation facilities [14]. This population is not served by functional sewerage systems; it utilizes on-site facilities [15, 16]. The on-site sanitation facilities are inadequate with the available ones facing pressure because of an ever-increasing population [14, 17]. Pit latrines are used widely in these areas, but they pose management and operational constraints [18, 19]^.^ The sanitation provision task does not end at the point of pit latrine construction. The objective of this study was to establish the relationship between user practices and sludge accumulation in pit latrines serving five low income neighbourhoods. An understanding of the factors determining FS accumulation rates is important when estimating the number of facility users and operational duration.

## Methods

### Study area

**Fig. 1:**
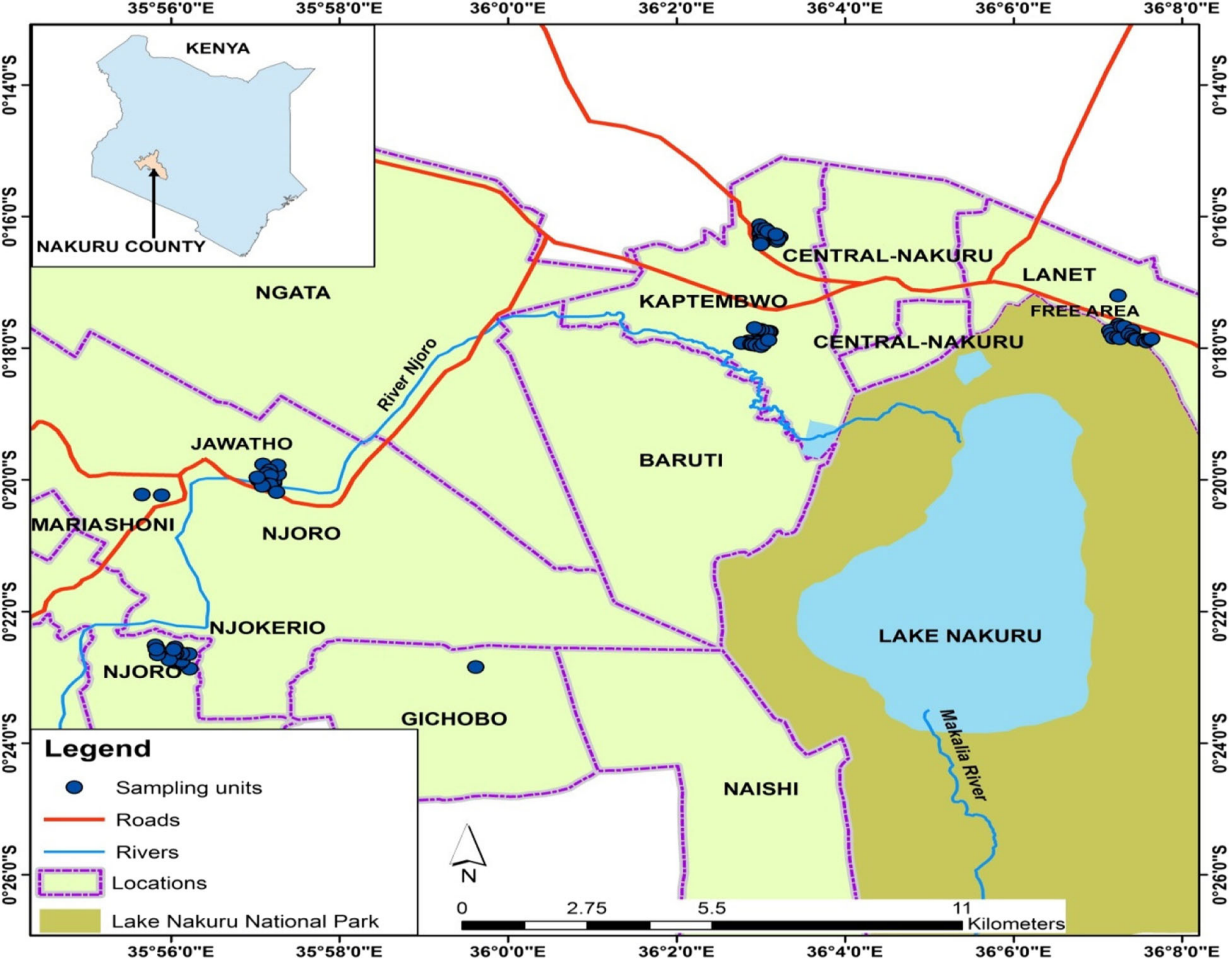
Study area showing the locations where the sampled pit latrines are located Source: Survey of Kenya Topographical Maps, scale 1: 50,000, using ILRI boundary Shape files: GIS ArcGIS 10.2

the study site was in five neighbourhoods within Nakuru county; Kaptembwo, Hilton, Free Area, Jewadhu and Njokerio (Fig. 1). Nakuru is the fourth largest urban centre in Kenya and has a population of about 307,990 inhabitants, of whom 190,000 live in low-income settlements [20]. These areas were chosen because of their unplanned households, lack of connection to sewerage infrastructure, low socioeconomic status and fast filling pit latrines. Also, the residents have an interest in improved sanitation services and some awareness activities are in progress. The main economic activities of the inhabitants are in the informal sector with irregular sources of livelihood. Recent statistics have shown that the areas had population expansion putting pressure on amenities. Moreover, the local authority has no pit latrine emptying programs and ‘FS’ treatment plants.

### Study design

the research applied a longitudinal study design. It involved a survey to provide insight into the pit latrine design and management practices plus their impact on facility performance across the five neighbourhoods. In addition, pit volume changes were monitored bi-monthly for 10 months. Data collected from the households and pit latrine was to inform management practices and fill-up within the study period only. In addition, management history of respective pit latrines was also documented.

### Sampling procedure

for inclusion into the study, the pit latrines had to meet all these criteria;

- Dry pit content: pit latrines without connection to water sources - this was to ensure that documented fill-up could be accounted for by faecal matter only and not influenced by ingress water from other purposeful sources such as connected bathroom. In addition, it meant that the biotic composition and air balance was in a natural state, hence no substantiated imbalance on biological processes
- Pit latrines serving more than one household daily - the property was to enable a substantial addition of the faecal matter into the vault on a daily basis and also account for consistent usage of the facility even if some users were absent for a certain period
- Pit latrines with contents not more than one metre from the drop-hole level - this meant that the sludge was close to the drop-hole where the measurements were being taken from hence reducing errors. Such facilities are almost reaching fill-up meaning they have had regular usage, which would inform the general objective of the study.
- Pit latrines which had not been emptied since construction - such pit latrines could be classified as good performing as they did not require regular emptying hence an assumption that they could support services over the entire study period. In addition, it meant that the users had been operating the facilities naturally, hence no interference with the structural balances within the vault

Pit latrines included in the study were selected purposefully based on the above inclusion criteria. Twenty households with pit latrines that satisfied the study inclusion criteria were selected each from Hilton, Jewathu and Njokerio, while 25 households were each selected from Free Area and Kaptembwo due to their larger areas and populations.

However, ten pit latrines could not be monitored until the end of the study due to logistical constraints and non-cooperative property owners. Therefore, 100 pit latrines formed the study sample for the survey. Fill-up rates of 73 out of the sampled 100-pit latrine were enumerated. The remaining 27 pit latrines were not included in calculations of sludge accumulation because of decommissioning before study completion, an uneven design which made an estimation of the actual volume impossible and voluntary withdrawal of property owners in the course of the study.

The neighbourhoods selected were mainly the low-income settlements in Nakuru County. Numerous low-income settlements and rural areas of developing countries have pit latrines with similar characteristics as in the case above. Therefore, the study is generalizable for dry shared pit latrines serving low-income non-connected settlements of these regions.

### Data collection

information on user activities were obtained using a questionnaire and an observation schedule focusing on user activities, design and management practices. Questionnaires were administered to the pit latrine owners and in cases where they were not available, individuals who had used the facility for a long period were surveyed. These would be tenants who had stayed in the premise for the longest period, compared to others and understood the facility’s operational history. These were done in five replicates alongside fill-up monitoring and comparative results were documented. The same respondents were surveyed in all subsequent sampling episodes. Waste disposal into the vaults were recorded as either purposeful or random. Purposeful disposal is the scenario where users intentionally use the pit latrine as the disposal point. However, random disposal is a scenario in which wastes are disposed into the pit latrine arbitrarily without pre-planning.

The pit latrine fill-up rates were measured using a digital laser range finder (Bosch PLR 25, Bosch GmbH) with a spirit level fixed to points within the latrine. The points were marked on the floors with physical scratches so as to enable accuracy during subsequent measurement from definite points in consecutive data collection events. In addition, tape measures and spirit levels were used for taking physical dimensions of the vaults. The total volume of the vaults was measured at the beginning of the study, followed by bi-monthly measurement of volume changes for a period of 10 months. The formulae below show the methods to fill-up determination;

i. **Accumulation** = (Initial vault volume-final vault volume)

*The sludge accumulation rate was calculated based on the average sludge accumulation against the total months for monitoring the study.

ii. **Daily fill-up rates** = (accumulation ÷days monitored) L
iii. **Accumulation per person per day (l/p/d)** = (daily fill-up rates ÷ number of users)
iv. **Annual accumulation rates** = (Fill up rates × 365 days) L
v. **Accumulation per person per year (l/p/yr.)** = (l/p/d × 365 days) L

Note; Pit total volume determination; the vault designs were in two forms, either circular or rectangular designs. Therefore; the volumes were determined using the radius or the dimensions of length, width and respective heights.

### Data analysis

normality testing of the fill-up data was done using Kolmogorov - Sminorv test and descriptive statistics (mean, standard error and standard deviations) of all the data were determined. The fill-up data was analysed in Minitab version 16 using descriptive statistics while the significance of variations of a fill-up rates of pit latrines within and across study location was analysed using a one-way analysis of variance (ANOVA). In addition, determinations of the specific study areas having differences in fill-up were analysed using the Fisher’s Exact Test. All the analysis was carried out at 95% CI.

### Safety precaution

fieldwork (fill-up monitoring) were done while wearing personal protective clothes and equipment. Instant hand Sanitizers and disinfectants were available for sanitizing the body, benches, laser ranger, GPS, and all appliances.

## Results

### Demographic characteristics

Table 1 and Table 2 present a summary of the findings. Table 1 describes the demographic characteristics of all the study respondents. Most of the respondents (63%) were female, because they were at home during the field visits. In addition, the highest proportion of the sampled age group ranged from 51 to 60 years, composition of the group was mainly the property owners. Only 1% of the respondents were uneducated and the highest proportion (37%) had primary level (basic) education. The majority (33%) of the respondents had an income ranging between 101 and 300 USD as illustrated in Table 1.

**Table 1:** Descriptive summary of demographic findings across all the study areas

Variable	Description	% of respondents
Gender	Male	37
	Female	63
Age of respondents (years)	≤20	12
	21–30	15
	31–40	10
	41–50	15
	51–60	27
	> 61	21
Highest education levels	Uneducated	1
	Primary	37
	Secondary	17
	Vocational	10
	Tertiary	35
Monthly income (USD)	< 100	5
	101–300	33
	301–500	15
	501–700	21
	701–900	17
	> 900	9

a.Total number of respondents-100

b.Data was collected at the beginning of the study and presented as averages for all the study units

**Table 2:** Structural properties of pit latrine and extent of sharing

Variable	Description	% of respondents
Latrine design (compartments)	1	25
(Observation)	2	31
	3	30
	4	10
	5	1
	6	2
	7	1
Duration of use (years)	< 5	5
(self-reported)	6–10	23
	11–15	25
	16–20	18
	21–25	12
	> 26	17
Sharing by individuals	< 5	6
(self-reported)	6–15	13
	16–25	17
	26–35	19
	35–45	21
	> 46	24
Sharing with families	< 5	31
(self-reported)	6–15	47
	16–25	18
	26–35	1
	36–45	2
	> 46	1
Type of Slabs	Timber	15
(Observation)	Cemented	78
	Earth	7

a.The total number of pit latrines-100

b.Data presented as averages for observations across all the study units

### Structural design and facility sharing

Table 2 describes the structural design of the pit latrines with a focus on factors that determine sharing (compartments) and cleanliness maintenance (slabs). All the pit latrines had iron sheet roofs and superstructure for privacy. It also describes the extent of sharing by individuals and across families.

The majority of the pit latrines (31%) had two doors leading into two compartments. The median use period was 15 years. However, it was noticed that 17% have been in operation for over 26 years without getting full. All the pit latrines were shared and the largest proportion (47%), were being shared by 6 to 15 families. The number of individuals sharing a pit latrine ranged from 4 to 56 people with a median of 23 individuals.

**Fig. 2:**
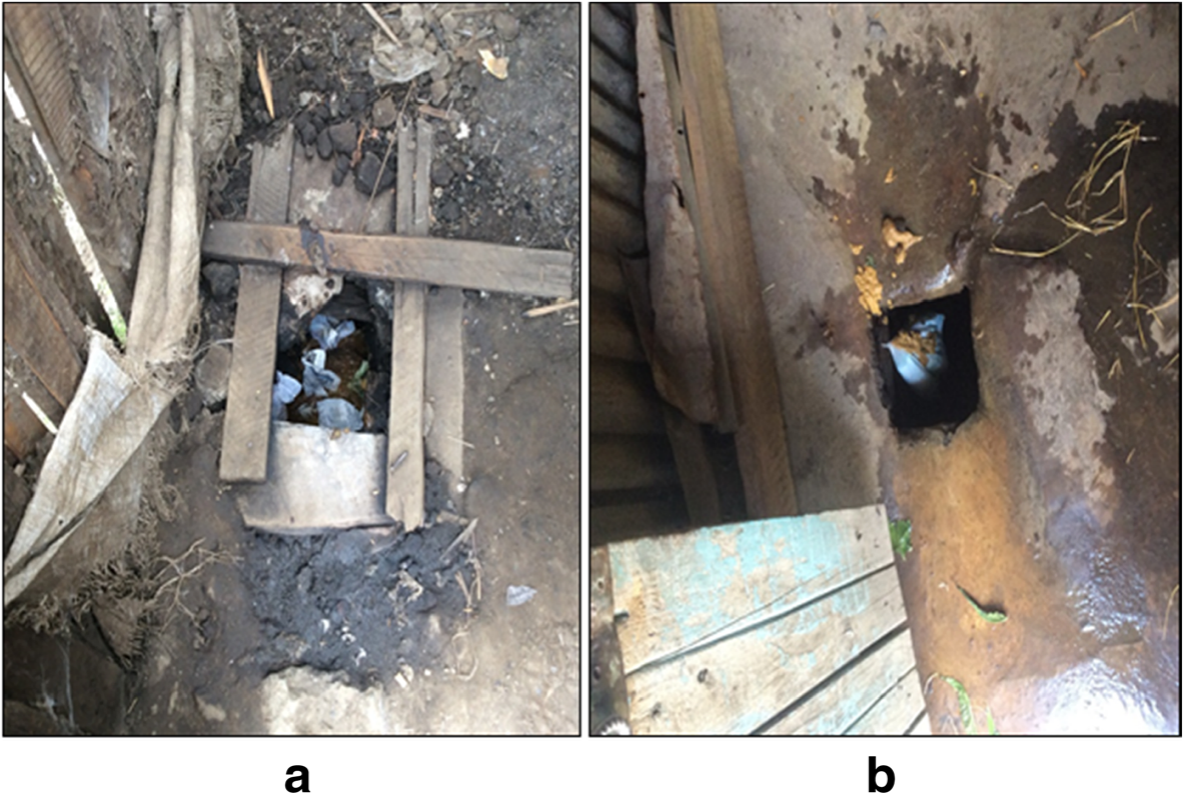
Solid waste disposal into the pit latrine (**a**-book pages & papers, **b**-diapers)

Anal cleansing materials varied with the largest proportion of households (47%) using tissue paper. As the study progressed, it was observed that household’s use of tissue paper increased with almost double proportions from 34 to 65% (Table 3). The type of anal cleansing materials does not only determine personal hygiene, but also affect sludge accumulation rates as some are slow degrading and others are non-degradable**.** Families provide the highest proportion of slab cleaning services (51%). Table 3 shows an increase in slab cleaning operation with every sampling episode**.** Observation of the vault and slab showed that 61% of the facilities had visible solid wastes (Fig. 2a and b). The materials included; polythene, papers, cardboard, diapers, clothes, plant leaves, plastic and glass bottles. Materials such as sanitary pads and diapers are purposefully disposed into the vaults whereas regular household wastes like papers and food remains are randomly disposed into the vaults.

**Table 3:** Consecutive ten-months pit latrines users’ operational activities

		Sampling Event (%)					
Variable	Description	1ST	2ND	3RD	4TH	5TH	Average
Anal cleansing materials	Tissue paper	34	41	43	52	65	47^a^
(self-reported)	Paper e.g. books	32	24	12	13	9	18
	Rocks &cobs	0	9	21	24	16	14
	Plant leaves	23	18	4	6	19	14
	Water	2	2	2	2	2	2
	Others e.g. Clothes	7	12	3	0	3	5
Slab cleaning	Families	26	39	49	62	79	51^a^
(self-reported)	Random cleaning	25	14	12	18	6	15
	Property owner	0	0	5	12	13	6
	Voluntary	35	29	28	18	12	26
	Workers	0	2	2	4	2	2

These activities were classified as the predominant operations and behaviour of households

Presented data are percentages

^a^ variable having the highest percentage

### Sanitation awareness and maintenance

Sanitation awareness is defined as the state of having been provided with information on the importance of pit latrine maintenance and the need for proper faecal matter disposal. The question was to document whether at any duration since they started using the facility, they had received any related information. The household survey showed that 45% of the residents had not received any form of sanitation maintenance awareness. Awareness creation varied and the government was cited as the most common awareness provider (21%) especially when inspecting facilities and during disease outbreaks. Other stakeholders who intermittently, provided sanitation related information included public health officials (15%), community based organizations and non-governmental organizations (8%), property owners (9%), volunteers and residents both accounted for 1%. The main facility cleanliness maintenance activity was slab washing or sweeping. Sixty-four percent of the respondents had regular unit cleaning schedules, whereas 36% did not have such programs. Regular unit cleaning schedules meant that users of a facility had personnel responsible for offering facility cleaning services within planned intervals of execution. The largest (51%) proportion of facility floor cleaning services was offered by families and the least (2%) proportion offered by workers.

### Pit latrine sludge accumulation rates

**Fig. 3:**
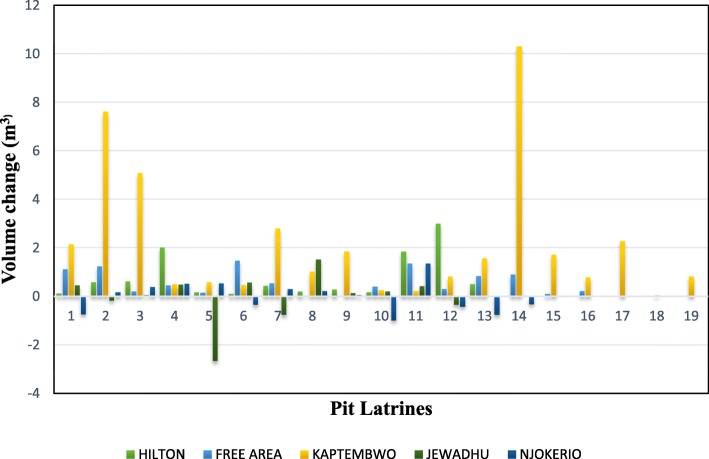
Vault accumulation per pit latrine (m^3^)

Sludge accumulation for the study period ranged from − 98 to 10,320 l and mean accumulation for all the pit latrines was 870 l. Accumulation varied within and across the study locations with Kaptembwo having highest and Njokerio recording the lowest rates (Table 4 & Fig. 3). Fill-up rates differed across the latrines with some recording accumulation increase while others had volume decreases as shown in Fig. 3.

**Table 4:** Pit latrine fill-up rates and annual sludge contribution per person

Fill-up Rates **(**m^3^/yr.)					Individual (l/p/yr.)	
Location	Mean ± SD	Min	Max	Mean ± SD	Min	Max
Hilton	0.78 ± 0.91	0.12	3.01	47.26 **±** 7.84	11.69	100.96
Free area	0.59 ± 0.50	0.01	1.49	34.10 **±** 20.94	16.62	75.39
Kaptembwo	2.16 ± 2.71	0.06	10.32	51.00 **±** 41.10	14.70	147.2
Jewadhu	0.24 ± 0.59	−0.76	1.54	31.30 **±** 14.70	14.8	59.51
Njokerio	0.01 ± 0.64	−0.98	1.37	47.26 **±** 24.79	21.93	101.36

Volume changes calculated per person per day and modelled to annual, 10 months sampling duration

**Fig. 4:**
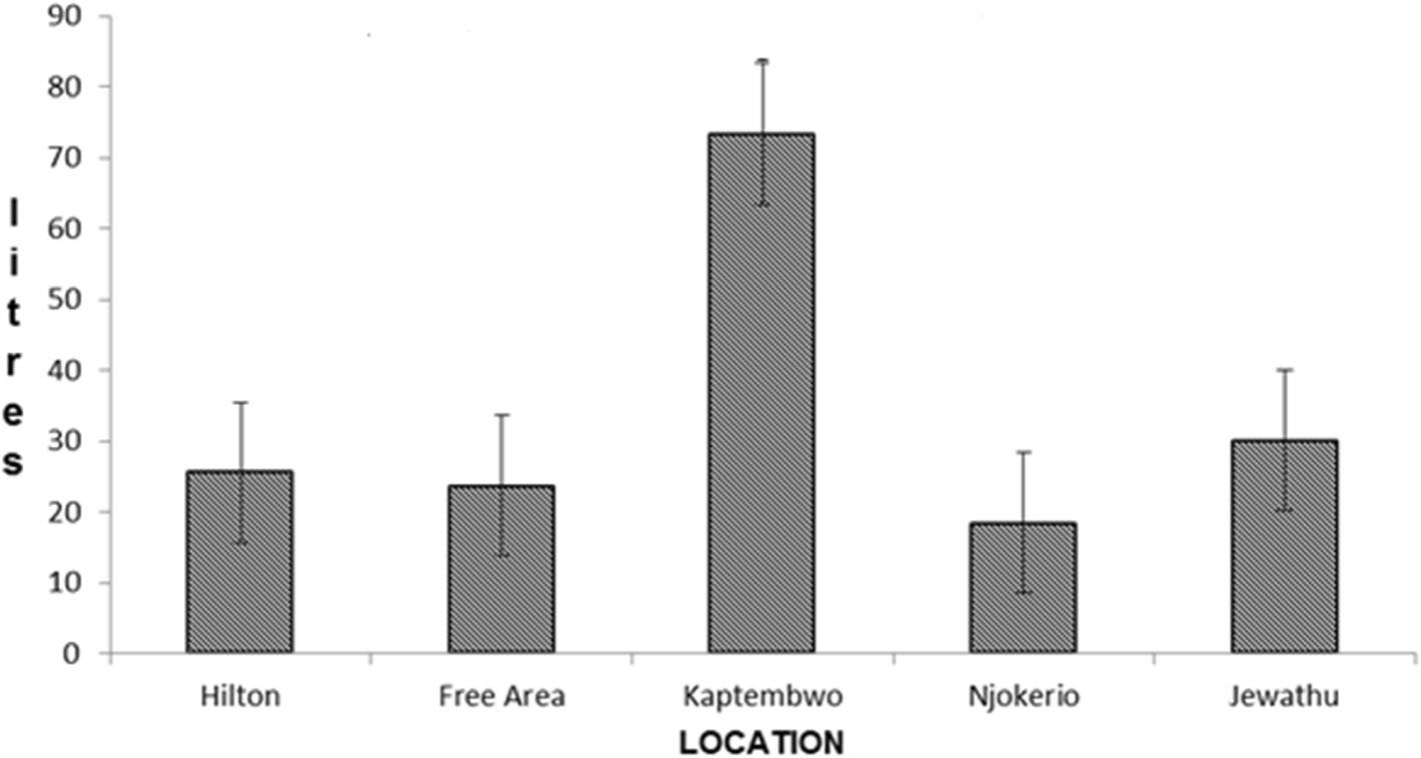
Mean annual contribution per person

The accumulation contribution per person ranged from 11.69 to 135.51 l per person per year, with a mean of 41.82 l per person annually (l/p/yr.). Noticeable is the high individual contributions reported in Kaptembwo (Table 4). The rates are relatively higher and could be explained by the documented disposal of wastes into the pit latrines within the area. The survey reported that solid waste and grey-water are disposed into the pit vaults. Figure 4 shows the mean and standard error bars of accumulation per location.

Longitudinal analysis on pooled data for the sites in each location indicated significant variability in the annual mean fill-up rates (m^3^ year^− 1^) across the five locations over the five sampling episodes (1-way ANOVA, F_4,1089_ = 14.80; *p* = 0.000). Confirmatory *post-hoc* Fisher’s exact test isolated Kaptembwo to record the highest annual fill-up rate 2.16 (±2.71) m^3^ year^− 1^. Hilton was significantly lower than Kaptembwo but higher than Free Area and Jewadhu in the fill-up rate per year. The lowest rate was observed in Njokerio with a mean rate of 0.01 (±0.64) m^3^ year^− 1^. The area which documented significantly higher fill-up rates had the highest rate of solid waste (70.83%) and grey-water (50%) disposal into the pit latrines. Therefore, the variations in user activities as documented by the survey justify the differences in fill-up rates of pit latrine within and across the study locations.

**Fig. 5:**
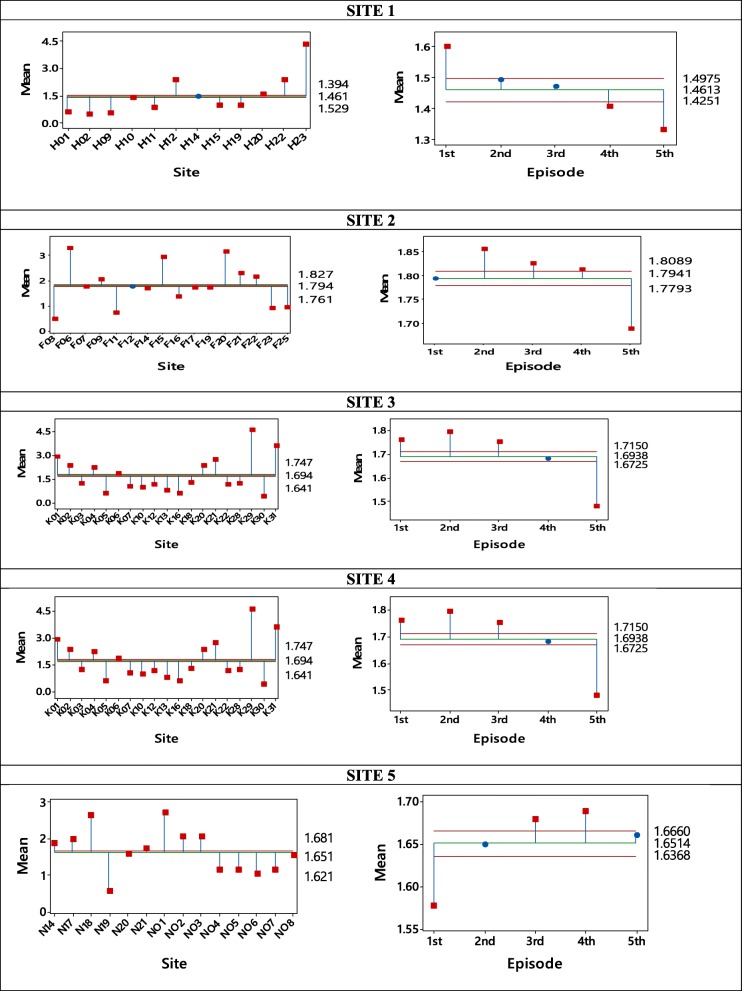
Spatial and temporal comparison within a location

Sludge accumulation trends within and across locations varied with each of the 5 sampling episodes. All the sampled pit latrines within a location showed erratic individual sludge contribution trends. Figure 5 shows the mean analysis of accumulation contribution per person over the study duration and the average sludge accumulation per site across the 5 sampling areas.

## Discussion

The findings are important to local sanitation delivery initiatives because the county and national governments have shown interest in the delivery of improved services through awareness and construction of improved facilities. In addition, the SDGs goal 6 has prioritized sanitation delivery to vulnerable populations such as undeserved low-income settlements. Data on pit latrine fill-up can be applied in developing sustainable pit latrine vault sizes and establishing emptying cycles. Defining user management activities and individual faecal sludge contribution are critical if pit latrines are to offer sustainable services. Most studies of pit latrines have focused on the superstructure and hygiene maintenance failing to consider what determines vault volume changes [21–23]. In this study, user management activities and characteristics of shared unlined pit latrines were measured as a total entity that included the number of users, use duration, daily activities and structural design. In addition, sludge accumulation rates were measured to understand how the user activities in the five sub-study locations varied and influenced accumulation rates. Linking these variables show that vault fill-up is not only attributed to the individual faecal sludge contribution but also management approaches. For example, pit latrine owners need to justify vault sizes based on the number of prospective users, their operational activities and expected duration of use which will also influence decisions on post fill-up management.

### User characteristics and design

Most of the respondents had attained primary education, an indication that they had basic education. The economic income of most of the respondents was relatively low (<USD300), given the extent of dependants within the households in the settlement. The findings are in congruence with results documented in 2013 by the Kenya Institute of Public Policy and Research Analysis (KIPPRA), explaining that low-income settlements are characterised by poor people who are likely to have low levels of education [24]. The majority of the inhabitants having a primary level of education are associated with low paying jobs, hence contributing to higher poverty levels. These characteristics would result in people constructing or using low-cost on-site sanitation services.

The studied units were simple pit latrines. Design and construction of the facilities had no plans regarding the numbers of use years and post fill-up management programs. Large proportions of the latrines are in locations that are inaccessible to emptying trucks. This necessitates emptying through manual methods (shovels, buckets, and spades) posing a health risk to emptying personnel, environment, and creating public health nuisance [17, 25–27]. Emptying would be faster and relatively cheaper as compared to abandoning a facility once full and constructing a new one. Such decisions have lower cost benefits in terms of resource allocation and encouraging open defaecation. According Eawag, an emptying cycle in Nairobi costs up to $130 [28], whereas construction of a new facility is approximately $430 [29]. Almost similar findings were reported in Dar es salaam that the cost of emptying was about $35 whereas constructing a new facility would be up-to 5 times higher [30]. Therefore, the cost-benefit of emptying contents is higher than the construction of new facilities [31, 32]. However, the scenarios within the study areas show that upon fill-up, pit latrines were either abandoned or decommissioned. Policies on the minimum acceptable shared pit latrine standards and operational management would provide significant impetus towards achieving the SDGs goals on sanitation and meeting the JMP standards [31, 33]. Highly-variable simple pit latrine designs are a major feature observed in many developing countries such as Kampala [34]. Provision of pit latrines without consideration of social, cultural, behavioural and gender aspects of sanitation facilities contribute to non-satisfactory services [35–37]. Generally, post fill-up emptying and management is not factored at design stages.

Single compartment latrines shared by more than one family do not only put pressure on facility use, but also limit basic sanitation maintenance. Such facilities significantly negate privacy and dignity of users, as people have to queue or wait outside as others use. Culturally, defaecation is viewed as a private affair; therefore, cases where one has to wait for his turn to use a facility greatly infringes on individuals psychosocial wellbeing. Building single compartment latrines could be attributed to the limited space and economic constraints within peri-urban low-income settlements [14, 33]. However, housing pressure associated with large population prioritizes housing settlements without factoring sanitation facilities. Nonstandard structural design and construction materials significantly affect hygiene and cleanliness of facilities, hence reduce sanitation quality. The case in Kenya is contrary to other countries in Africa with a minimum acceptable standard for a sanitation facility; South Africa (VIP latrine), Malawi (Blair VIP latrine) and Botswana (Double Vaulted VIP latrine). Kenya has no infrastructure designs on acceptable basic pit latrine standards [38]. It adopted a WHO task force recommendation of VIP latrine standards which have not been publicised leading to limited stakeholder awareness [34]. Therefore, the owners’ socioeconomic status widely determines the design of the facilities, a fact that resonates with other findings in most urban settlements of developing countries [39]. There is a need to actualize the designs of the latrines, create awareness programs supporting their development and support the minimum acceptable shared pit latrine designs.

The median number of people sharing a latrine was 23. It is higher compared to the numbers (10 people) reported in Kampala [40]. However, it is lower compared to those reported in Kibera, Kenya as 150 people per pit latrine according to the UN-World Bank report in 1997. When more families are sharing then it could influence people to resort to open defecation especially when there are long queues during peak periods of facility use (early morning and night). The JMP (2015) defined unimproved sanitation as a pit latrine without a slab, flush or pour flush to elsewhere, bucket, hanging latrine, shared sanitation or no facility [41]. None of the studied facilities met the WHO standards on improved sanitation. Case studies conducted in Kibera and informal settlements of Kisumu, Kenya have confirmed that shared facilities are appropriate sanitation provision options in high population settlements [38, 39]. The findings of this study suggest that shared facilities play an important role in sanitation service delivery to residents of low-income settlements [22, 26, 34]. This information concurs with a study in Kampala, which reported that informal settlements populations far exceed the sanitation infrastructure capacity hence communally shared facilities are common [42].

High urbanization rates in the low-income settlements lead to increased waste generation [43]. Solid and liquid waste handling services are relatively insufficient, because local authorities do not provide the services, but have contracted refuse collection to private handlers. These have associated costs; hence residents opt for disposal into pit latrine vaults or the environment. Grey-water disposal facilities are not provided and end up being discarded in the environment with a large proportion poured into the pit latrines. Water disposal affects sludge moisture content and gaseous balance, therefore, interfering with aerobic and anaerobic degradation processes [44]. Pit latrines and the environment form part of the alternative point of waste disposal in these areas. Similar inefficient wastes handling in similar settlements have been reported in many regions of the world, including Blantyre [45], Kolkata [46] and EThekwini Municipality [47].

Households in the study area showed little attention and emphasis to provision of anal cleansing materials inside a facility. Similar findings indicating significantly low emphasis and high ignorance on the importance of anal cleansing materials in the WASH provision have been reported in previous studies [48–51]. Lack of anal cleansing materials affects personal hygiene and has a strong association with faecal contamination and disease spread [52]. In addition, use of solids and slow degrading solids for anal cleansing contributes to high organic load and encourage faster accumulation [4]. When on-site facilities fill-up faster, they lead to the management burden of either emptying or building a new pit latrine, health risks, and occupational hazards associated with sludge disposal [53]. In addition, menstrual products adversely affect fill-up rates in cases where sanitary pads are disposed in the pit latrines [54].

To achieve maximum performance of sanitation facilities, there is a need of linking hardware (structural design and technology) and software (hygiene practices, policy, and education) components [52, 55]. However, in this case there is limited provision of the necessary information, yet education and awareness are important in the primary health provision. Although knowledge and awareness should be provided to the design and construction personnel on most appropriate facilities and to users on management and operations of the facilities, there is no such evidence. Awareness would provide an informed decision, hence better services and enhanced facility performance. A large proportion of household had not yet received sanitation awareness and this could explain the reported low levels of facility cleanliness maintenance and cases of solid waste disposal into the vaults [56]. The willingness to pay for sanitation maintenance services may present constraints due to the general low-income [18, 23].

Results from this study indicate that if economically sustainable services and public health protection is to be attained, then these issues which directly affect user operations must form the basis of policy recommendations. Further noted in this study is that the designs must factor the extent of sharing and operational management. Other activities such as waste disposal and the inconsistent number of users would not support long term and safe delivery. However, when the users’ operational characteristics are factored at design stage, then decisions linking information and structural components would lead to improved sanitation delivery.

### Sludge accumulation rates

The mean sludge accumulation rates of 41.84 l/p/yr. were relatively moderate compared to the WHO recommendation of 40 to 60 l/p/yr. and up to 90 l/p/yr. where dry cleansing materials are used. The accumulation was within the range reported by WHO in the 1950s indicating a mean annual fill-up of 40 l in dry pit latrines characterised by solid materials and other waste disposals [57]. However, they are lower than data from Brazil [58] and Besters camp South Africa that documented an average of 90 and 69.4 l/p/yr. [25]. On the contrary, studies in South Africa reported relatively lower accumulation rates of 24 l/p/yr., but indicated a 50% increase in cases where solid wastes are thrown into the vault [59]. The fill-up rate documented in this study indicates that a pit latrine of 2.5 m^3^ would serve up to five people for 12 years. These rates can be used for designing vault sizes applicable for specific populations. Further, modelling of such ratios would inform programs of vault emptying cycles. The survey data showing individual contribution can be used alongside modelled accumulation rates to design specific use period.

Significant differences in fill-up rates can be attributed to the differences in the user practices and management activities as presented in the survey. User practices account for the characteristics and make up of faeces that determine degradation of the faecal sludge [9]. Varying geographical locations and socioeconomic incomes may have contributed to the variations. In addition, solid and liquid wastes have a potential of suppressing content degradation and encouraging vault content accumulation. The varying household practices across the study areas as reported in the survey justify the differences in fill-up and performance of the pit latrines. The variations in fill-up of pit latrines across these study areas are consistent with previous studies [40].

The findings of this research give evidence of the importance of both user operational management and structural components of pit latrines in determining pit latrine service duration. To increase pit latrine performance and efficiency, then the design of vault sizes must be justified by the intended number of users and use duration [60–63]. Moreover, all the stakeholders have to be educated and provided with appropriate information supporting various stages of sanitation service delivery from facility design, operational phase and post fill-up management. If the stages are handled in isolation, then the sanitation outcome may not be beneficial to users, property owners and the environment. Linking information and awareness regarding appropriate facility development and operations would contribute the progress in sanitation ladder [56, 64, 65]. Such measures would ensure efficient, economically viable and sustainable pit latrines hence protection of public health and the environment.

### Limitations

This study was designed to factor variation in seasons. However, the country experienced a drought hence there were no significant variation in rainfall volumes across the months of the study. However, it would be possible to have different fill-up rates in the rainy and dry seasons. The findings of the study are applicable within the context of Kenya and other low-income settlements within the sub-Saharan Africa. Moreover, these findings can form a basis for comparison studies in other low-income settlements. Facility physical environment was not investigated hence future studies ought to establish soil permeability around each pit latrine and possible sinking of true bottom.

## Conclusion

The study intended to document pit latrine user management practices and associated fill-up variations. Though the user activities varied within and across the study areas, one of the sub-study locations had a significantly higher fill-up rate. It documented the highest number of individuals sharing facilities and waste disposal into pit latrines which is a phenomenon linked to higher fill-up rates that necessitate regular emptying, abandoning of facilities, decommissioning of structures and the use of un-hygienic full pit latrines. This leads to significant public health risks, environmental degradation, and economic constraints to users, property owners and the government. Shared sanitation facilities played a significant role in the sanitation service provision, but evidently present numerous management constraints. The study recommends that property owners, local communities and authorities need can embrace such facilities, but establish efficient operational and post fill-up management programs. Property owners and artisans need to understand applicable standard pit latrine designs. In as much as the user practices determine fill-up rates of pit latrines, the link between information (awareness and training) and structural designs would result in significant improvement in pit latrine performance hence better sanitation services.

Findings from this study indicate that sustainable sanitation services can be attained if the nexus between the number of facility users, design, vault sizes, operational management and post fill-up activities are factored at all stages. Sludge accumulation rate is an issue that should be considered at sanitation planning stage. Pit latrine design, construction and supporting budgets should factor post fill-up management and must have a minimum acceptable standard. If the pit latrine vault sizes, structural designs, operational management and number of users are factored in urban sanitation planning then there will be lower cases of environmental contamination associated with open defeacation, overflowing pit latrines and facility use pressure as a result of sharing across many households. These will lead to lower fecal contamination in the general environment hence public health protection. Further studies should model fill-up of pit latrines under different socioeconomic set ups, diets, soil types and across seasonal variations. Moreover, there is a need to establish microbial diversity that determines degradation rates, hence form a basis of bio-remediation of vault faecal sludge.

## Abbreviations

ANOVA: Analysis of Variance
CBOs: Community based organisations
CI: Confidence Interval
GIS: Geographical Information Systems
GPS: Global Position Systems
ILRI: International Livestock Research Institute
JMP: Joint Monitoring Program
NACOSTI: National Council of Science and Technology-Kenya
NGOs: Non-governmental organisations
SDGs: Sustainable Development Goals
VIP: Ventilated Improved Pit latrine
WHO: World Health Organisation
